# Beyond the machine: masculine ecologisation in *Kadaisi Vivasayi* (2022) and *Malayankunju* (2022)

**DOI:** 10.3389/fsoc.2026.1881494

**Published:** 2026-07-16

**Authors:** Vishnu Jain, I. Ajit

**Affiliations:** School of Social Sciences and Languages, Vellore Institute of Technology, Chennai, India

**Keywords:** eco-feminism, ecological masculinities, masculine ecologisation, sensory ethology, south Indian cinema

## Abstract

This research investigates the “Crisis of Sensibility” inherent in modern malestream masculinity, framing it through the ecofeminist lens of the “logic of domination”—a patriarchal framework that reduces the living world to a mere backdrop for human achievement and extraction, while justifying the dual subordination of nature and “others.” By synthesising Hultman and Pulé’s ADAM-n model of Masculine Ecologisation (the structural roadmap) and Baptiste Morizot’s Sensory Ethology (the somatic toolkit), the paper proposes the Etho-ADAM-n model. This analytical synthesis provides a framework for men attempting to exit the ‘machine’ of Industrial/Breadwinner Masculinity—an identity defined by an “illusion of control” and “crude aggressiveness.” The study uses two South Indian films to illustrate this transformation. Through a descriptive cinematic analysis of the films *Malayankunju* (2022) and *Kadaisi Vivasayi* (2022), the study tracks how film form facilitates a sensory re-engagement with the biotic community. Rather than presenting a resolved ethical state, the research identifies these narratives as fragile thresholds of vulnerability sparked by environmental rupture and agrarian precarity. Ultimately, the transition models a shift from patriarchal mastery towards a precarious state of ecological belonging.

## 1 Introduction

The contemporary ecological crisis is often viewed not only as a material issue but also fundamentally as a “Crisis of Sensibility.” This crisis in the relationship with the living world is described by the philosopher Baptiste Morizot (2022) as a “tragic impoverishment of the modes of attention” that humans pay to other-than-human beings. This leads to a reduction in the affects, precepts, and practices that connect us to the Earth (Morizot, 2022). Over the last four centuries, Western Modernity has created devices and systems that relieve us of the need to pay attention to ‘alterities’, leading to what lepidopterist Robert Pyle calls an “extinction of experience.” This is marked by a low sensitivity to the living beings that inhabit the Earth with us (Pyle and Louv, 2011). The direct contact with the vitality of the Earth is replaced by the sterility of the “Machine.” The machine logic is historically grounded in the motif of “the machine in the garden,” a concept developed by Leo Marx (1964). It refers to a device that represents a “crude, masculine aggressiveness,” which suddenly intrudes upon and destroys a pastoral idyll to prove manhood through dominance (Marx, 1964). Within the construction of masculinity, this manifests as the “hermetic” masculine body—an identity traditionally characterised as sealed, impermeable and hard that reduces the environment to a mute backdrop for human achievement (Waldby, 1995; Mosse, 1996; Hearn, 2014). Masculinities are dynamic, plural and socially constructed, reflecting “a confluence of multiple processes and relationships” across varied groups and institutions (Gardiner, 2002). Hegemonic masculinity serves as the dominant configuration of gender practice, defining the standards that men are expected to live up to if they are to be valued (Connell, 1995).

This hegemonic model stands as the central focus of ecological feminism, which seeks to dismantle the structural causes of environmental and social harm. Gerta Gaard explains that ecofeminism’s fundamental idea is that “the ideology which authorises oppressions such as those based on race, class, gender, sexuality is the same ideology which sanctions the oppression of nature” (Gaard, 1993). For scholars in this field, concepts like ‘machine’ or the ‘hermetic body’ are more than just symbols and metaphors. They are tangible results of a patriarchal mindset that treats traditional male values as the valid standards for all human beings. According to Karren Warren (1987), this system relies on value-hierarchical thinking, which views the diversity of life through a vertical lens, where status is granted only to those perceived to be at the top. This unequal system is kept alive by forcing life into two opposing sides, like setting logic against emotion or culture against nature. Thus, falsely separates elements that are actually interconnected.

To dismantle this structure, the project of Ecological Masculinities emerges as a vital subset and extension of ecofeminism. This framework seeks to transition the masculine self from Val Plumwood’s (2002) “Rational Economic Man” to a sensing animal who acknowledges his ecological embeddedness. The hegemonic masculine identity is codified in the theoretical category of Industrial/Breadwinner Masculinities, which serves as the primary driver of ecological decay. The particular category describes an identity “enmeshed with industrial scale extractive processes and services reliant on energy-intensive, profit-consolidating, ecologically destructive and fossil fuel dependent processes” (Hultman and Pulé, 2018). In this model, environmental costs are overlooked in favour of capitalist gains. Plumwood (2002) describes the “Rational Economic Man” as a “mean, lean, tough machine, shorn of compassion” that operates with a cold “economic rationalist imaginary.” These subjects prioritise self-interest at the expense of others, where ‘others’ include women, nature, non-humans and the marginalised (Plumwood, 2002; Sahu and Choudhury, 2025). By viewing the ‘others’ as a mute backdrop for human achievement, this mindset drives the formation of industrial/breadwinner masculinities. This model socialises men from an early age to become mechanistic ‘human-doings’ (performers) who must suppress their own human being-ness and capacity for relational care (Morizot, 2022). To protect this detachment, men use rationality as an armour to distance themselves from their radical deep reliance on the living world (Hultman and Pulé, 2018). In the South Indian context, this ‘hermetic’ shield is strengthened by a logic of domination that is not merely technical, but socio-cultural. Drawing on Mukul Sharma (2024), this paper argues that caste identity functions as an “archaeology of untouchability”—a sensory regime of touch, smell and feel. This system shapes the masculine subject’s connection with the shared soil, translating the crisis of sensibility into a refusal to recognise a mutual vulnerability with nature and the Dalit “other.”

This paper highlights the films *Malayankunju* (2022) and *Kadaisi Vivasayi* (2022) as cinematic responses to harmful environmental norms. However, the protagonists occupy different ideological spaces. In *Malayankunju*, Anikuttan’s detachment from the environment reflects his internalised casteism. His refusal to share space with Dalit neighbours creates a barrier to biological community. Conversely, Maayandi in *Kadaisi Vivasayi* represents a model of ecological belonging. He has already dismantled the industrial machine and practices a diplomacy of interdependence that heals the communal divide. Through the joint celebration of land, he embodies an identity free from discriminatory norms.

To track these transitions, I propose the Etho-Adam-n model, an analytical synthesis where Hultman and Pulé’s (2018) structural roadmap for identity (Adam-n) is activated by Baptiste Morizot’s (2022) somatic toolkit (Sensory Ethology). By careful examination, the paper treats cinematic form as a sensory engine which captures the protagonists’ physical transformation from mechanised mastery to relational care (Morizot, 2022). Instead of presenting a resolved ethical state, the research views these narratives as fragile thresholds of vulnerability triggered by environmental disruption and agricultural instability. This approach allows us to see cinematic forms as a sensorium of cultural self-renewal, leading towards a state of multispecies ubuntu—the realisation that “I am what I am because we, the living, are what we are.”

## 2 Literature review

The existing scholarship on *Kadaisi Vivasayi* (2022) and *Malayankunju* (2022) provides a robust foundation across the fields of eco-cinema, sociology and trauma studies, though these films are rarely examined through a unified gender-ecological model. The study of Ecological Masculinity functions as the primary theoretical anchor, which is a transdisciplinary field that seeks to resist the identity-shaping economic structures of industrial capitalism. The foundational work by Hultman and Pulé (2018) established a framework that draws on four streams: ecofeminism, deep ecology, masculinist politics and feminist care theory. It moves beyond the extractivist Industrial/breadwinner model and the reformist eco-modern masculinities to ecological belonging and relational care. This transition aims to dismantle the “Rational Economic Man,” a figure that prioritises instrumental self-interest at the expense of the living world (Plumwood, 2002).

Recent studies on South Indian Cinema have documented a major shift from superhuman heroes to “ordinary men” with human flaws. Krishnan and Balakrishnan (2023) claim the protagonist of *Malayankunju* represents “subordinate masculinity,” whose identity is formed by unresolved traumas and a mechanical way of living, which conceals emotional dejection. This sociological shift is further explored in trauma narratives, where the landslide in *Malayankunju* turns into a “second coming of trauma,” freeing the protagonist from a cycle of sadomasochism and leading him to empathy (Remya and Rukmini, 2023). This sociological change sets a baseline for following a change from mechanised detachment to ecological belonging.

Similarly, *Kaidaisi Vivasayi* has been analysed in Tamil Cinema as an instance of sustainability communication. Researchers have examined the film in relation to Sustainable Development Goals, specifically SDG2 and SDG15 (Jeeva et al., 2024). The traditional farming method of the protagonist Maayandi has been contrasted with the harmful “machine” of industrial farming. The film’s emphasis on seed science is highlighted by Mohamed Kurzith Khan and Aysha Rizwana (2026), as the protagonist Maayandi resists hybrid seeds as a form of resistance against a market system that prioritises corporate profit over biophilic health. These studies position the film as a critique of industrial modernisation as opposed to Maayandi’s traditional husbandry and spiritual link to the land. In addition, the film is also noted for its depiction of interspecies diplomacy where Maayandi’s legal defence for the dead peafowls subverts the anthropocentric legal norms.

A critical thread in the current research explores how environmental issues are inseparable from social hierarchies. Karthika (2022) documents how caste identity functions as a “logic of domination” in the domestic front, infiltrating private spaces. To move beyond surface associations, this study draws on Mukul Sharma (2024), who theorises ‘eco-casteism’ as an “archaeology of untouchability.” This framework identifies a visceral sensory regime of touch, smell, and feel that structures the masculine subject’s relationship with the soil and the wider biological community (Sharma, 2024). In *Malayankunju* (2022), Anikuttan’s “crisis of sensibility” is a somatic manifestation of this regime; his refusal to share space or amenities with Dalit neighbours is a sensory refusal to coexist within the same biological community. According to Karren Warren (1987), this mindset is driven by a “logic of domination,” a patriarchal conceptual framework that uses vertical hierarchical thinking to justify the subordination of whoever is considered lower. This aligns with Gerta Gaard’s premise that the ideology authorising the oppression of marginalised groups is the same that sanctions the oppression of nature (Gaard, 1993).

Despite the depth of this existing scholarship, a critical academic void remains in the synthesis of social critique with the sensory and somatic metamorphosis of the masculine subject. First, there is a lack of cross-cultural theoretical application. Most foundational eco-masculinity scholarship remains focused on North American fiction, leaving a gap in applying this framework to the caste-inflected context of South Indian Cinema. Second, the existing research treats sociological shifts, structural policy and psychological trauma as separate tracks. There is a lack of unified analysis that synthesises social frameworks with a sensory reawakening. Finally, while researchers note that characters undergo transitions, the field lacks rigorous step-by-step documentation of how cinematic form—using spatial audio, sync sound and visual rhythms—facilitates the somatic transformation of the masculine subject from a model of dominance to a model of interdependence. This research fills these gaps by proposing the Etho-Adam-n model to track these transitions through specific film signs.

## 3 Materials and methods

The study primarily uses a qualitative and descriptive cinematic analysis as its primary methodological approach. The analytical procedure involves repeated close viewings of the selected films to facilitate deep interpretative exploration of narrative structures, visual aesthetics and character arcs. This approach utilises descriptive scrutiny to translate technical film signs into evidence of character metamorphosis. By treating cinematic form as a sensory engine, the analysis moves beyond symbolic reading to observe technical execution of ecologisation.

To perform this analysis, the films are divided into sequential narrative units to systematically track the protagonists through the stages of the Etho-ADAM-n framework, where sequences and scenes are analysed to identify the narrative junctures where protagonists show specific masculine traits or undergo identity shifts. Finally, the research is structured around a comparative case-study lens, contrasting a ‘process model’ of transformation with a ‘template model’ of ecological belonging.

### 3.1 Theoretical framework

This paper applies the Etho-Adam-n model, a theoretical synthesis combining masculine ecologisation with philosophically enriched ethology. The model functions as a theoretical bridge where the two primary pillars perform a distinct division of labour: Hultman and Pulé’s (2018) Adam-n precepts serve as the structural roadmap, defining *what* shifts in the masculine identity, while Baptiste Morizot’s (2022) sensory ethology provides the somatic toolkit, defining *how* technical filmic signs execute that shift.

The first pillar engages with Hultman and Pulé’s (2018) ADAM-n model precepts—Awareness, Deconstruction, Amendment and Nourishment—which is a staged ‘exit-politics’ from the ecocidal norms of Industrial/Breadwinner masculinities. Within this section, it is necessary to explain the structural logic of each stage:

- Awareness(A) involves educating the self to move beyond “eco-chambered judgements”
- Deconstruction(D) involves a critical analysis of the “logic of domination” and extractive industries.
- Amendment(A) involves taking accountability and responsibility, making amends to the Earth.
- Modification(M): necessitates choosing a pathway towards relational care.
- Nourishment(n): represents the configuration of new masculinities that celebrate biodiversity and interspecies community.

The second pillar integrates Baptiste Morizot’s (2022) sensory methods, redefining ethology as an “enriched style of attention” to the signs and meanings of the living world. This toolkit treats cinematic form as a sensory engine, utilising concepts such as “vibratory vigilance” and “interspecies diplomacy” to document character metamorphosis. Central to this is the “adjusted consideration,” a practice where the subject treats other life forms as “alien kin,” with their own unique existence and rights. By combining these structural and sensory frameworks, this paper observes the masculine subjects’ transition from a hermetic masculine body to a “Panimal” body, which is porous and sensing (Morizot, 2022).

## 4 Discussion

### 4.1 . Case study 1: *Malayankunju* (2022)—shattering the machine

Anikuttan in *Malyankunju* (2022) serves as a local embodiment of the “Rational Economic Man,” an identity that is tied to technical productivity and social detachment (Plumwood, 2002). He leads a clockwork lifestyle as an electronic technician, using his workspace as a shield to isolate himself from his neighbours and natural environment. Anikuttan’s initial relationship with nature is one of neglect, where he sees the environment as a mere backdrop to his work. His complicity in the “crisis of sensibility”-tragic impoverishment of attention to the living world–is highlighted by his professional relationship with the resort owner (Morizot, 2022). The resort owner, Francis, acts as a critical foil representing the extreme of industrial masculinity by clearing a real forest to build a resort that relies on artificial sounds to attract tourists. In a casual exchange at the resort, the owner characterises his synthetic soundscape as ‘aural pleasure’, serving as a visceral illustration of the crisis of sensibility. This sarcastic talk, where the owner mockingly agrees with Sumesh that “people like you have cleared all [the forests],” exposes the cynical heart of the Capitalocene (Prabhakar, 2022, 00:30:40). This era is defined by Jason W Moore not merely as an economic system but as “a system of power, profit and re/production in the web of life” that organises nature into a passive backdrop for capital accumulation (Moore, 2017). The owner performs what Baptiste Morizot calls a total “extinction of experience,” replacing a living ecosystem with an engineered simulation. As the technician, Anikuttan, is the one who facilitates this for profit by setting up the synthetic soundscape.

*Malayankunju* (2022) unfolds during the peak of the monsoon season in the hilly topography of Idukki district in Kerala. Surrounded by an atmosphere of approaching doom, characterised by the constant rumble of thunder and incessant rain, the film presents a world that is fundamentally unstable. This instability is rooted in the region’s high vulnerability to landslides, a threat that the film portrays as an inevitable catastrophe. These disasters do not arise from natural causes alone but rather from anthropogenic interventions that have made the Idukki district a major hotspot for landslides (Jones et al., 2021). Spatial modelling reveals that the main cause of these landslides is land-use changes, in particular, the deforestation and conversion of forest lands into farmland. Studies reveal that out of all the landslides that happen in the region, 59.38% occur in the modified lands where plantations play a role (Jones et al., 2021). In addition, quarrying also accelerates geological mass movements by disregarding natural slope and terrain conditions. Factors like these reflect the systemic neglect of the Western Ghats’ ecological stability.

In this unstable world, Anikuttan retreats to his electronics workspace, which serves as a hermetic shield, maintaining an illusion of control over his environment. This workspace is a physical manifestation of what Mukul Sharma (2024) defines as the “archaeology of untouchability”—a visceral sensory regime of touch and sound that structures the subject’s alienation from his community and the living world. Cinematographer Mahesh Narayanan technically constructs this detachment through a claustrophobic visual rhythm, utilising tight close-up shots to box Anikuttan into his circuit boards. This visual enclosure reinforces his status as a ‘Rational Economic Man’ whose caste-based isolation leads to a tragic “extinction of experience” with the natural environment (Morizot, 2022). However, the film’s broader mise-en-scène subverts this enclosure by incorporating a pervasive blue visual palette into the frames to foreshadow the rain-driven disaster (Jayadeep, 2022). This creates a state of dramatic irony where the audience sees nature already intruding upon his caste-segregated world, even as the protagonist remains sensorily within his boxed space. Anikuttan’s mechanised self is further constructed through aural insulation. Despite being hyper-sensitive to sound, he uses sound as a weapon of detachment. He plays devotional songs at full volume to aggressively drown out the geopolitical messages of his community. The primary target is the high-frequency cry of Ponni, the neighbour’s infant daughter. His refusal to tolerate this Dalit child’s voice illustrates a ‘crisis of sensibility’ where caste prejudice functions as a somatic defence against the noisy nature of the biological community. This aural suppression represents the “ethics of the charioteer,” where technical reason is used to subdue the noisy nature of the living world (Morizot, 2022). This particular sensory alienation is best shown in an early scene where Anikuttan intentionally throws away beef curry at a local eatery. He does this because the bowl was shared with fellow diners whom he considers the ‘other’. This act shows that caste is not simply an idea; it is a physical defence mechanism. The protagonist retains the purity of the body by violently rejecting shared biological resources. By viewing common food as a source of ritual pollution, the film highlights how Anikuttan’s crisis of sensibility starts in his gut and hand.

Anikuttan’s journey is defined at its outset by a profound failure of Awareness(A), the first precept of the ADAM-n model. This stage requires a high degree of personal reflexivity and openness to the biotic community (Hultman and Pulé, 2018). Instead, Anikuttan remains blind to the impending danger signs and dismisses government warnings about landslides as publicity stunts. This denial of ecology is one characteristic feature of the “Rational Economic man” who assumes that his technological isolation has insulated him against any kind of ecological vulnerability. He refuses to move to the shelter home, specifically because he detests sharing provisions with other castes. The caste system, deeply embedded in his beliefs, functions as an unnatural social structure that separates him from his community as well as the shared soil. This internalised superiority manifests most violently when he uses a casteist slur against his neighbour, Suni, following an argument about his baby’s cry disturbing his technical work. This aggression functions as a defensive performance of his rigid hegemonic masculine identity. It is constructed to mask the chronic hurt and unaddressed trauma surrounding his father’s suicide, which occurred after Anikuttan’s sister eloped with a man from a marginalised community.

The origin of Anikuttan’s adult state of mechanised detachment is found in a pivotal flashback scene. This scene depicts a young Anikuttan witnessing his father, Radhakrishnan, and a group of friends shooting a lactating wild boar to protect their crops, a moment that depicts a stark state of human self-enclosure. The dialogue highlights the “logic of domination” used to justify the act:

Friend: “This will be a police case. It has given birth recently. Its children will be somewhere.”

Radhakrishnan: “Only we know what we are going through. It was ruining everything we were cultivating.” (Prabhakar, 2022, 00:34:19–00:34:26).

This instance serves as Anikuttan’s initiation into a breadwinner mindset, where manhood is proven through the nullification of animal sentience. By witnessing the men shoot a lactating mother boar, young Anikuttan is exposed to a deontological framework, where the moral duty to protect human livelihood serves as a ‘misappropriated opportunity’ for ruthless action and sadistic behaviours (Anto, 2025). From a deontological perspective, the act is scrutinised not just for its results but for the moral intention and violation of the duty to respect a ‘subject of life’ (Anto, 2025; Regan, 1983). The boar and the offspring have an intrinsic right to exist independent of human utility. This early trauma shatters his nascent “Ethics of Care,” an orientation rooted in interconnected self which defines morality through relational responsibility and empathy. This initiation teaches him that true manhood involves adopting a “sado-dispassionate” mode of reason (Plumwood, 2002). Anikuttan uses detached rationality as a shield to deny empathy or identification with “the Other,” whether the other is a nursing boar or his Dalit neighbour. His masculinity is further shaped by the instrumentalism displayed in the scene, where a complex ecosystem engineer is reduced to a mere obstacle to production (Plumwood, 2002). The act reflects an ecological arrogance that prioritises short-term agricultural security over long-term forest health. The indiscriminate killing of such ecosystem engineers destabilises the food chain, depleting the prey base for larger predators and leading to a feedback loop of human-animal conflict (Ayyappan, 2025). This shows how the illusion of control that is intrinsic to his father’s masculinity lays the foundation for the environmental anxiety and the catastrophic landslide that Anikuttan faces later as an adult.

The landslide acts as the catalyst for the active Awareness(A) stage of the model, which is violently forced upon Anikuttan as nature physically breaches his technological world. This catastrophic event destroys his house and electronic workshop—a literal “Machine in the Garden” that represented his masculine attempt to isolate himself from the natural world (Marx, 1964). The landslide sequence functions as a visceral “ethogram,” a catalogue of behavioural sequences documenting Anikuttan’s transition from a mechanical ‘human doing’ to a sensing animal (Morizot, 2022). The lengthy episode contains minimal dialogue, relying instead on the immersive aural environment of the landslide. The visual design depicts a subterranean space so narrow that only one person can crouch and crawl. This claustrophobic environment physically breaches Anikuttan’s hermetic masculine armour, forcing a wet porosity with mud and soil (Hearn, 2014).

This leads into the Amendment stage(A). It is defined as an opportunity for course correction, where an individual takes responsibility for the social and environmental costs of their previous lifestyle (Hultman and Pulé, 2018). While trapped underground, Anikuttan undergoes an ecological awakening. He activates what Morizot calls “The Panimal” body, recovering ancestral animal senses and instincts to survive and navigate the debris (Morizot, 2022). Cinematographer Mahesh Narayanan documents this ‘animal ascent’ through tight close-up shots of Anikuttan’s frantic eyes and faltering screams. The realism of the subterranean enclosure is sonically reinforced by Dolby Atmos spatial audio, which positions sounds directly above the head of the audience. This acts as a sensory bridge that physically traps the viewer inside the mud-filled abyss with Anikuttan. It ensures that the baby’s cry is not just a noise but a spatial coordinate that guides the protagonist’s animal ascent. The sequence relies on a single-source torchlight held by the actor himself to illuminate the darkness (Filmkopath, 2022). This raw, unmediated encounter strips away his identity as a technical performer, forcing him to inhabit his animal self. Anikuttan owns his mistakes and realises the fact that his refusal to move to the shelter house directly caused the death of his mother and his own predicament.

During this struggle, a pivotal moment of Deconstruction(D) occurs through a dream sequence of his father, Radhakrishnan. This stage involves a critical analysis of the mechanisms of domination that structure a subject’s life and their relationship with the environment (Hultman and Pulé, 2018). Trapped forty feet underground and courting death, he encounters a hallucinatory apparition of his father. Their dialogue serves as the moment where his mechanised detachment collapses:

Anikuttan: “What happened, Dad? Where are we?”

Father: “Cannot you sense a rotten smell of land? It was a landslide. Our house is now deep underground.” (Prabhakar, 2022, 01:09:23–00:09:40).

His father serves as an equaliser, dismantling Anikuttan’s caste-based worldview by reminding him that all beings ‘sleep equally’ in the soil. He instructs Anikuttan to save Suni’s baby Ponni, whom he detested.

The writer-cinematographer Mahesh Narayanan conceptualised the baby’s cry as a “gift from nature” designed by evolution to force human attention (Filmkopath, 2022). Guided by the high-frequency cry of Ponni, he survives in the course of saving her. The baby’s cry is no longer a noise that destroys his concentration but a sensory indicator that guides him through the subterranean void. Anikuttan’s sudden focus on the infant’s wailing manifests what Morizot defines as “neotenia”—a primal tenderness towards the young that signals an animal empathy much more profound than simple human sentiment (Morizot, 2022). By choosing to save the child, he moves into the Modification stage (M), finally exchanging his mechanical isolation for a commitment to relational care. However, his empathy here is largely performative, born of the necessity of surviving the mud-filled abyss. While this act serves as the stage of an ethical transition, the film defers the resolution to the very end, leaving the long-term depth of his change ambiguous. It is only through his silent smile in the children’s ICU that his psychological armour seems to fully collapse, yet the narrative never explicitly confirms if this “Panimal” reawakening can withstand the prevailing social pressures of the caste order once he returns to his everyday environment.

### 4.2 Case study 2: *Kadaisi Vivasayi* (2022)—The diplomat of interdependences

While *Malayankunju* tracks a violent rupture, director M. Manikandan opens *Kadaisi Vivasayi* with a deliberate unhurried cinematic rhythm that utilises a wide canvas to visually decentre the human subject from the very first frame. The introductory sequence uses extra-long shots to construct an expansive view of the landscape, in which vast agricultural plains stretch towards distant mountain ranges and scattered settlements (Rajan and Jayalakshmi, 2025). These images emphasise the immense presence of the land, highlighting its scale and enduring nature. Within this grand eco-spectacle, the human inhabitants appear almost minuscule, woven into an imposing fabric of the natural world. This visual strategy immediately grounds the film in a state of Awareness(A), as it portrays an octogenarian protagonist, Maayandi, who has already performed a radical Deconstruction (D) of industrial masculinity. Maayandi represents a model of ecological belongingness, portraying a masculine subject who has succeeded in overcoming the crisis of sensibility. Being the “last farmer” in the village where everyone has sold their lands to the financiers, Maayandi becomes the direct challenge to the extractivist logic of modern-day development. By turning down financial offers, he shows an awareness of the land’s inherent value beyond its usefulness to people. This stands in stark contrast to his neighbours, who have accepted the principles of Capitalocene. In this system, the living world is rationalised and treated only as a passive backdrop for capital accumulation, which has led the villagers to sell their ancestral land (Moore, 2017). Maayandi, however, embodies a successful departure from this hegemonic model of mechanised detachment.

Maayandi serves as a model, but the narrative is aware of the agricultural instability and labour crisis that complicate this idyllic vision. The old feudal ties, which historically bound agricultural workers to severe food insecurity, have been disrupted by governmental programmes like the Public Distribution System (PDS) and MGNREGA. These initiatives have diverted the local labour force (Kumar, 2022). While these changes secured the right to dignity and food security for the marginalised, they left the traditional practitioner physically alone. Maayandi’s solitary labour, depicted in the scene where he unsuccessfully tries to recruit a group of women for his field, is portrayed not as a romantic choice but as a desperate effort to resist the ongoing loss of traditional knowledge amid the changing Capitalocene.

The story of Maayandi rests upon a fundamental Awareness(A) which directly questions the model of the “Rational Economic Man,” a subject who operates under an illusion of total autonomy while treating the environment as a mere storehouse of resources (Plumwood, 2002). In a seamless display of Awareness, Maayandi tastes cattle fodder in the market to ensure its worthiness for his cattle, for which he was mocked by the seller. When questioned by the merchant, Maayandi provides a direct explanation for his sensory engagement: “I cannot just feed my cattle without testing it” (Manikandan, 2022). This act can be seen as a rejection of consumer forgetfulness where humans interact with nature only passively. By personally verifying the quality of the feed, he accepts his own well-being is inextricably tied to the health of his livestock.

Building up on this foundation, Maayandi performs Deconstruction(D) through a radical rejection of industrial shortcuts. He refuses hybrid seeds and chemical pesticides, linking the health of the seeds to the vitality of the community. When the merchant offers high-yielding seeds which require constant re-purchasing, Maayandi is surprised. Their exchange serves as a critique of a system that prioritises profit over the natural cycle of life:

Maayandi: “How could a fruit tree not have seeds?”

Seller: “It’s a white man’s invention. The fruit does not have seeds.” (Manikandan, 2022, 00:12:25–00:12:30).

Maayandi recognises this invention as an act of instrumentalism, where nature is treated as a passive, sterile commodity rather than a living partner. His practice of agriculture is one of “husbandry,” which emphasises interaction, inhabitation and reciprocity with the land (Allister, 2004). This contrasts sharply with the profit-oriented attitude of his neighbours.

In a pivotal scene, Maayandi discovers three deceased peafowls in his cropland and provides them with a ceremonial burial (Manikandan, 2022). His act of burying the dead peafowls is a practical application of “Adjusted Consideration.” This philosophical concept, as defined by Morizot, is a form of ethical sensitivity where a human constantly adjusts their attention to do justice to the unique life of another being (Morizot, 2022). The burial serves as the Amendment stage(A) of the model, where he takes responsibility for the costs the living world has incurred. Maayandi does not consider the birds pests or property under patriarchal state law. Rather, he sees them as “alien kin”—beings that share an evolutionary history with humans, but that retain an essential mystery (Morizot, 2022). His burying of the birds is an act of reciprocity, an acknowledgement that even in death, these creatures are residents who belong to the land as much as he does. He is subsequently arrested and placed in judicial remand for 15 days, the physical price for his ethical commitment to his non-human kin.

Maayandi’s legal trial is reflective of the “crisis of sensibility” that pervades modern-day institutions. In this case, the court process and police conduct themselves in accordance with a hegemonic rationality—a mode of thinking that reduces nature to an instrumental lens, viewing the peafowl as evidence or property belonging to the state (Plumwood). When the magistrate orders a police constable to water Maayandi’s crop, the constable’s failure to tend to the crop illustrates the epistemic remoteness of the system. The legal system lacks the sensory connection required for husbandry; it cannot sustain the life Maayandi has cultivated. This highlights the system’s carelessness and detachment from the biological reality of the land. Maayandi represents the Modification (M) stage, through his practice of husbandry—a style of attention that modifies one’s deed to support the life of others. His refusal to treat the crop as a simple commodity is visible during this legal battle, where he acts as an interspecies diplomat.

Maayandi is pulled from his role as a solitary practitioner of husbandry to the public and institutional sphere through both communal necessity and state intervention. He acts as a Janus-faced diplomat, mediating directly with the needs of the living soil while simultaneously acting as a spokesperson for interdependences before human institutions. In court, he challenges the court to recognise its responsibility and dependency on nature. This is clearly seen when he confronts the magistrate, arguing that his freedom is tied to the survival of his crops:

“Money comes and goes. We sowed seeds for our deity, and they have already sprouted. They need to be watered. Are they not living things? Wouldn’t they die? By holding one life here, you are killing a thousand lives” (Manikandan, 2022, 01:10:26–01:10:40).

Maayandi’s assertion that the paddy plants possess life demonstrates a degree of sensory alertness that the legal system fails to accommodate. This is in line with Carol Gilligan’s (1982) ethics of care, which says that moral problems are solved through an understanding of contextual relationships and reciprocal responsibilities rather than through the application of formal, abstract laws.

Maayandi demonstrates what Morizot (2022) defines as “vibratory vigilance,” the constant sensory alertness to the signs and deeds of other life forms. In *Kadaisi Vivasayi* this sensory turn is not just a thematic suggestion but a technical requirement achieved through the director’s use of sync sound technology. The film captures live, surrounding audio directly in the fields of the Usilampetti region rather than utilising studio dubbing. Thus, the production intentionally reduces the psychological distance between performers and the audience (Rajendran, 2022). This technical rigour forces a state of “vibratory vigilance,” where the viewer must attend to the land as a living, breathing subject rather than a mere backdrop for human drama (Morizot, 2022).

### 4.3 Synthesis: towards multispecies ubuntu

The synthesis of these cinematic narratives signifies the final shift to the Nourishment(−n) stage of the ADAM-n model. The protagonists move from simply surviving to actively cultivating and sustaining a new, ecologised masculinity defined by relational care. In this stage, both men transition from “human doings”—valued strictly for their utility within a capitalist system—to “human beings” who accept their place within a wider biological community. This transformation happens through what philosopher Baptiste Morizot terms an “incandescent alloy” of human identity, which melds pragmatic human reason—Anikuttan’s electronics skill and Maayandi’s agricultural knowledge—with a primal sensing empathy that recognises our common ascent with all living beings (Morizot, 2022).

In *Malayankunju*, this synthesis finds concrete expression through the hospital plot line, where Anikuttan appears fragile and bandaged, but he is finally at peace. The appearance of Anikuttan at the crib of the baby Ponni and his smile towards her indicate that the previous barricades of caste and technical isolation have been torn down. He moves beyond the “machine in the garden” to rejoin the biological community, choosing to become a guardian for the life he saved (Marx, 1964). While he has resolved his individual “crisis of sensibility” by acknowledging his mutual vulnerability with other lives, his empathy remains survival-driven. A descriptive scrutiny of this final scene—his bandaged body and weary smile-suggests that his resolution remains precarious.

Similarly, *Kadaisi Vivasayi*, concludes with the temple festival, where Maayandi acts as a considerate “guardian of interdependences.” Despite being ignored by the government and forced to sell his land, he fulfils his ecological duty by providing the first grain needed for the communal ceremony. This act is a powerful moment of communal healing because it brings together all the different caste groups in a society to celebrate a harvest that sustains the entire biotic community. By providing the first harvest, Maayandi performs an act of interspecies diplomacy that temporarily blunts the rigid boundaries of the Brahmanical social order, proving that a man’s true success lies in sustaining his community and the soil rather than seeking industrial profit. The depth of this nourishment is validated through the indirect transformations Maayandi facilitates in the younger generation and the state. This is most vividly captured in the collaborative labour between the police constable and Maayandi’s grandson. The constable, having undergone an amendment through his physical irrigation, finds a stress-free life in the soil. His confession that the 2 hours spent in the field are his “only peaceful time” signifies an exit from the institutional masculine armour of the state enforcement into a state of ecological belonging. By tending the field together, these men forge their own ‘incandescent alloy’ embodying the village elder’s wisdom that “Only when one breaks free of their thoughts they become Gods” (Manikandan, 2022). This symbolises breaking free of the rigid anthropocentric thoughts of domination.

Ultimately, both films lead to a state of multispecies ubuntu—the profound realisation that “I am what I am because we, the living, are what we are.” By the end, Anikuttan and Maayandi have reconnected to the real world. Even if Anikuttan’s resolution remains precarious, his specific moment of self-realisation alongside Maayandi’s steadfast husbandry proves that men can be woven back into the web of life, finding wholeness as members of a biotic community.

## 5 Conclusion

The journey of Anikuttan and Maayandi serves as practical templates for masculine ecologisation, a process defined as relationality in action. These films document a transition away from the ‘ethics of the charioteer’, where reason is used to subdue personal passion and treat the natural world as a passive backdrop for human progress (Morizot, 2022). While Anikuttan serves as a case study for the transformation required to exit a mechanised clockwork lifestyle, Maayandi serves as a template for steadfast defence of ecological husbandry against a modern industrial machine. Initially, Anikuttan is trapped by an illusion of control that separates him from his community, whereas Maayandi’s neighbours exemplify this detachment by viewing the land solely as a commodity for industrial profit.

By the end of these narratives, protagonists embody the “ethics of the diplomat,” adopting a practice of ‘adjusted consideration’ to protect the interdependences that sustain their respective territories (Morizot, 2022). This shift is achieved through the practical application of ADAM-n Model, as they navigate the internal deconstruction and behavioural modification necessary for masculine ecologisation. This evolution culminates in a state of ‘multispecies ubuntu’, where their identity is inextricably linked with the well-being of the entire biotic community. The broader implications of these stories suggest that true masculine success lies in protecting the glocal commons and tending to local ecological needs that ultimately impact the health of the entire planet. This transition proves that men must remain woven into the fabric of life to resolve the “crisis of sensibility.” Ultimately, these films offer guidance, moving modern man away from dominance and back toward a life of shared care, mutual vulnerability and ecological belonging.

## Data Availability

The original contributions presented in the study are included in the article/supplementary material, further inquiries can be directed to the corresponding author.
